# Single-chip photonic transceiver based on bulk-silicon, as a chip-level photonic I/O platform for optical interconnects

**DOI:** 10.1038/srep11329

**Published:** 2015-06-10

**Authors:** Gyungock Kim, Hyundai Park, Jiho Joo, Ki-Seok Jang, Myung-Joon Kwack, Sanghoon Kim, In Gyoo Kim, Jin Hyuk Oh, Sun Ae Kim, Jaegyu Park, Sanggi Kim

**Affiliations:** 1 Electronics & Telecommunications Research Institute, 218 Gajeongno, Yuseong-gu, Daejeon, 305-700, Korea

## Abstract

When silicon photonic integrated circuits (PICs), defined for transmitting and receiving optical data, are successfully monolithic-integrated into major silicon electronic chips as chip-level optical I/Os (inputs/outputs), it will bring innovative changes in data computing and communications. Here, we propose new photonic integration scheme, a single-chip optical transceiver based on a monolithic-integrated vertical photonic I/O device set including light source on bulk-silicon. This scheme can solve the major issues which impede practical implementation of silicon-based chip-level optical interconnects. We demonstrated a prototype of a single-chip photonic transceiver with monolithic-integrated vertical-illumination type Ge-on-Si photodetectors and VCSELs-on-Si on the same bulk-silicon substrate operating up to 50 Gb/s and 20 Gb/s, respectively. The prototype realized 20 Gb/s low-power chip-level optical interconnects for λ ~ 850 nm between fabricated chips. This approach can have a significant impact on practical electronic-photonic integration in high performance computers (HPC), cpu-memory interface, hybrid memory cube, and LAN, SAN, data center and network applications.

Silicon photonics technology can open a new dimension in data interconnects for future computers and communication systems, with unprecedented bandwidth based on the cost-effective silicon photonic/complementary metal–oxide–semiconductor (CMOS) platform^1^,^2^,^3^,^4^,^5^,^6^,^7^,^8^,^9^,^10^. By utilizing monolithically-integrated chip-level optical inputs/outputs (I/Os) instead of electrical I/Os, the electronic-photonic convergence in silicon electronic chips can enable revolutionary changes in computer architecture, data communications and telecommunications^7^,^11^,^12^,^13^,^14^.

In recent years, the performance of silicon photonic devices and various levels of photonic integration have shown remarkable progress^15^,^16^,^17^,^18^,^19^,^20^,^21^,^22^,^23^,^24^,^25^,^26^,^27^,^28^,^29^,^30^,^31^,^32^,^33^,^34^,^35^,^36^,^37^,^38^,^39^,^40^. Continued improvements in device performance and increased levels of integration are being pursued with the goal of full utilization of optical interconnects based on the monolithic integration of photonics with electronics. However, efforts to implement silicon photonic devices as viable optical I/Os in electronic chips still face major obstacles, such as large device size and low operational efficiency, high power consumption, and so on.

Also, there is a substrate incompatibility issue with conventional silicon electronic chips when applying photonics for chip-level optical interconnects^25^. Most of the main silicon photonic devices, such as waveguide-type modulators, photodetectors (PDs) and wavelength division multiplexing (WDM) devices, need a light-confining environment such as silicon-on-insulator (SOI) substrates, whereas most electronic chips are based on bulk silicon substrates. Therefore, transitioning to the electronics based on SOI substrates for the electronic-photonic convergence is not very practical or realistic at the moment.

Furthermore, realizing a compact chip-level light source is a serious issue for practical implementation of a silicon photonic I/O scheme on a silicon electronic chip. At present, due to the lack of a practical light source based on Group IV elements, flip chip-bonded or packaged lasers based on III–V semiconductor devices are being used as external light sources, to feed silicon modulators on SOI-based platforms to complete a photonic transmitter. This does not accomplish monolithic integration as well as a chip-level complete photonic I/O set. The exception may be the reported silicon hybrid lasers integrated on SOI wafers, which have an active region based on III–V materials^41^,^42^,^43^,^44^,^45^,^46^.

Due to above major problems, a new photonic I/O scheme which can overcome these obstacles and ease the transition to the electronic-photonic integration at silicon chip level, is of great interest.

In this work, we propose a new scheme for chip-level photonic I/Os, based on monolithically integrated vertical photonic devices on bulk silicon, which increases the integration level of PICs to a complete photonic transceiver (TRx) including chip-level light source. This approach can realize a compact self-contained optical I/Os, while providing solutions to substrate-compatibility, and integrated light source with low-power consumption in silicon optical interconnects. We demonstrate a prototype of the single-chip photonic TRx based on a bulk silicon substrate for λ ~ 850 nm optical interconnects at high data rates, proving that this approach can offer compact low-cost I/O solutions for electronic-photonic integration. In particular, this scheme can be also utilized in the realization of 3D optical interconnects with 3D-stacked ICs, as well as in immediate applications of network transceivers.

## Results

### Scheme, design and fabrication of photonic TRx chip

New photonic integration scheme, a single-chip photonic transceiver is comprised of monolithic-integrated vertical-illumination type Ge-on-Si photodetectors (Ge V-PDs) and direct-modulation light sources, vertical-cavity surface emitting lasers on a bulk silicon substrate (VCSELs-on-Si). The integration of Ge V-PDs is based on CMOS-compatible process on a bulk-Si wafer, and that of VCSELs-on-Si is based on the transplanted epitaxial film and the device fabrication on the same bulk Si wafer as the Ge V-PDs.

**Figure 1:**
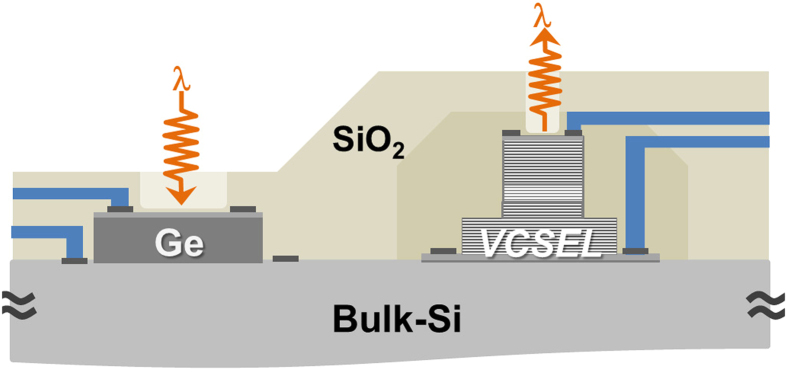
Schematic diagram of monolith-integrated TRx devices on bulk silicon wafer. This can be an integrated photonic I/O platform for major electronic chips based on bulk silicon, or compact single-chip photonic transceivers for network applications.

Figure 1 shows a schematic diagram of monolithic-integrated vertical TRx devices on a bulk silicon substrate, which can be used as a photonic I/O platform for optical interconnects, applicable to electronic chips based on bulk silicon, or as compact cost-effective single-chip transceivers for network applications. Here, a Ge V-PD is used as a monolithic-integrated chip-level photonic receiver (Rx) on a bulk silicon substrate. This configuration has many advantages including substrate compatibility with electronic chips, low-loss in coupling and packaging due to larger clear aperture, and demonstrated high-performance which can readily replace conventional III-V compound semiconductor PDs^35^,^37^,^40^. The direct-modulation VCSELs-on-Si is employed as a monolithic-integrated photonic transmitter (Tx) on the same bulk-silicon wafer with the Ge V-PDs, and can solve the compact chip-level light source problem, which is one of the main obstacles to implementing silicon-based optical interconnects at present. VCSELs, emitting at various wavelengths, are well-established, compact, reliable, low-power laser light sources and widely used in all levels of optical interconnects^47^,^48^,^49^,^50^,^51^,^52^,^53^,^54^. In this scheme, high performance VCSEL devices based on recent progress can be transplanted and utilized on a bulk silicon substrate, as an integrated compact reliable light source, without much degradation of device performance. This approach can enable practical implementation of the chip-level silicon-based optical interconnect.

In the following, we demonstrate a compact bulk silicon photonic TRx chip. The paper presents the design of the vertical photonic TRx devices integrated on a bulk silicon substrate, chip fabrication processes, and characterizations of the integrated devices.

**Figure 2:**
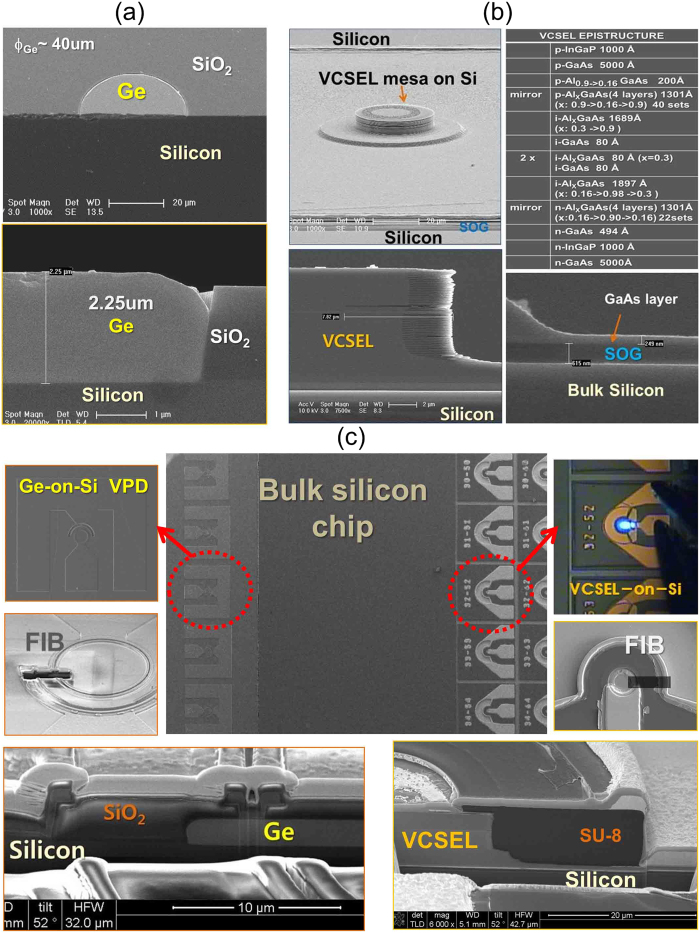
(**a**) Tilted side-view and cross-sectional SEM images of the 2.25 μm-thick germanium layer, selectively epitaxially-grown into a 40 μm-ϕ__Ge_ mesa window for fabrication of a vertical-illumination type PD on bulk silicon. (**b**) Epi-structure for the λ ~ 850 nm VCSEL, and top-view and cross sectional SEM images of an integrated VCSEL mesa on a silicon substrate, after ICP and wet etchings, and oxidation process of device fabrication. The Figure shows a ~600 nm-thick SOG between the bulk silicon substrate and the GaAs contact layer of the VCSEL epitaxial film. (**c**) Top-view SEM image of the fabricated photonic transceiver silicon chip with monolithically-integrated TRx devices, Ge-on-Si V-PDs and VCSELs-on-Si (top-center figure), and enlarged top-view SEM image and 52 degree-tilted cross sectional FIB SEM images of a Ge-on-Si V-PD with a 40 μm-ϕ_Ge_ mesa and a 2 μm-W_Ge_ (left figures), and a microphotograph of a light-emitting VCSEL-on-Si with a 32 μm-ϕ_VCSEL_ under operating current, and 52 degree-tilted cross sectional FIB SEM images (right figures).

The Ge V-PD, as a photonic Rx in a silicon chip, is based on a simplified structure and a selective expitaxial growth (SEG) by reduced pressure chemical vapor deposition (RPCVD). The device structure was adopted that allowed a simplified fabrication process without chemical mechanical polishing (CMP) or doping for germanium^37^,^40^. The Ge V-PDs are designed in array form and placed on the left side of a chip, leaving the right side of the chip dedicated to the process area for the integrated direct-modulation light source. Germanium epitaxial layers were selectively grown into predefined open windows for PD mesas with various diameters ranging from 20 μm to 100 μm on the 6” Si (100) wafer with SiO_2_ layer deposition, using RPCVD at 380 °C/650 °C without additional thermal annealing. Germanium up to ~4 μm thickness can be selectively epitaxially-grown with good quality. A cap layer of 0.1 μm-thick in-situ boron-doped poly-silicon provided a heavy and uniformly doped layer for the p-type contact without ion implantation process. Figure 2(a) shows a tilted side-view and cross-sectional scanning electron microscopy (SEM) images of ~2.2 μm thick (W_Ge_) Ge selectively-grown into a mesa window area with 40 μm-diameter (ϕ_Ge_) on a bulk silicon substrate. The device fabrication was performed based on a CMOS compatible process. After integration of the Ge-on-Si V-PDs, a SiO_2_ layer was deposited to protect the integrated V-PDs on the chip.

For the optical Tx on the same bulk silicon chip, the monolithic integration process of the light source was started with a substrate lift-off VCSEL epitaxial film on the dedicated region of the chip by wafer bonding^55^,^56^. Here, the spin-on-glass material (SOG) was coated on the silicon chip to bond a small piece of the VCSEL epi-chip in the opened window area for the Tx, on the right side of the chip.

Any type of VCSEL epi-structures designed for emitting wavelengths within the effective absorption wavelength range of germanium, from λ ~ 650 nm to λ ~ 1600 nm, can be used. Here, for the prototype, we used a VCSEL epi-structure designed for λ ~ 850 nm, containing 3 GaAs quantum wells with an etch-stop layer grown on a GaAs substrate, as shown in Fig. 2(b). The bonding process was performed with pressure in a furnace chamber at ~200 °C. The separation of the epitaxial film from the GaAs substrate on which it was grown was accomplished by etching process with NH_4_OH solution.

Device mesas with a diameter (ϕ_VCSEL_) ranging from 27 μm to 40 μm were defined with the substrate-removed epitaxial film on bulk silicon. Laser devices are designed in array form. Figure 2(b) shows top and cross sectional SEM images of a VCSEL mesa defined on a bulk silicon substrate (top figures), and after an oxidation process defining a device active aperture of ϕ_aa_ diameter was performed (bottom figure). The figure also shows a ~600 nm-thick SOG between the silicon substrate and the GaAs contact layer of the epitaxial film. Additional fabrication of the laser device was carried out including planarization with a SU-8 for ~10 μm-tall VCSEL mesas and metallization. The planarization process used here with a thick SU-8 can be replaced with a SiO_2_ passivation process or a polyimide process. A window opening process for the protected Ge V-PDs during the VCSELs-on-Si fabrication process was performed for device characterization. A SiO_2_ passivation layer can also be used as an anti-reflection coating optimized for λ ~ 850 nm.

As mentioned before, the extension to longer wavelength VCSELs-on-Si in the λ ~ 1310 nm or λ ~ 1550 nm range for example, can be straight forward by using properly-designed VCSEL epi-wafers based on an InP substrate with various wafer bonding processes.

In Fig. 2(c), the top-center figure shows a top-view SEM image of the fabricated prototype of a photonic TRx chip, which is comprised of monolithic-integrated Ge V-PDs and VCSELs-on-Si on the same bulk silicon substrate. The left figures show SEM images of a Ge-on-Si V-PD with a 40 μm-ϕ_Ge_ mesa and a 2 μm-*W*_*Ge*_, and top-view and cross sectional SEM images of an integrated PD prepared by focused ion beam milling (FIB) process. The right figures show a microphotograph of a light-emitting VCSEL-on-Si with a 32 μm-ϕ_VCSEL_ under the operating laser current, and SEM images of the VCSEL-on-Si prepared by FIB process.

### Device characterizations

**Figure 3:**
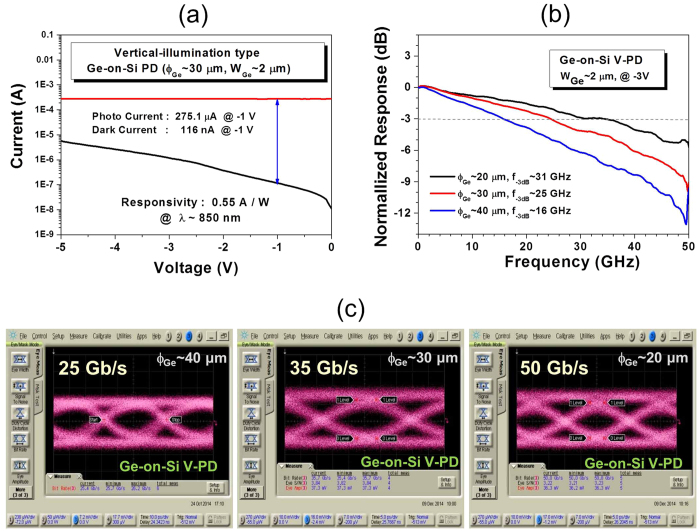
(**a**) Typical I–V characteristic of the fabricated Ge-on-Si PD with a 30 μm-ϕ_Ge_ and *W*_Ge_ ~ 2 μm for λ ~ 850 nm. (**b**) The measured frequency responses of integrated Ge V-PDs with a 40 μm-, 30 μm-, and 20 μm-ϕ_Ge_ (**c**) On-chip eye diagram measurements of the integrated Ge V-PDs with a 40 μm-, 30 μm-, and 20 μm-ϕ_Ge_ at 25 Gb/s, 35 Gb/s and 50 Gb/s data rate, respectively.

Monolithic-integrated Ge V-PDs were characterized with current-voltage (I-V) measurements, and on-chip measurements of eye diagrams at high data transmission rates. Figure 3(a) shows a typical experimental current-voltage (I-V) characteristic for a Ge V-PD with a 2 μm-*W*_Ge_ and a 30 μm-ϕ_Ge_. The light was delivered to the device by a lensed multi-mode optical fiber probe. The dark current (black line) is as low as ~116 nA at −1 V. The red line indicates the photocurrent measured under an illumination of λ ~ 850 nm, and the responsivity (*R*) is 0.55 A/W.

The bandwidths of the integrated Ge V-PDs were measured by impulse response with a Menol femtosecond pulse laser and Agilent DCA^57^. Figure 3(b) shows the experimental frequency responses of the fabricated Ge V-PDs with a *W*_Ge_ ~ 2 μm and a ϕ_Ge_ ~ 20 μm, 30 μm, and 40 μm with a -3dB bandwidth, f_-3dB_, of ~31 GHz, 25 GHz, 16 GHz, respectively. The non-return-to-zero (NRZ) pseudo-random bit sequence (PRBS) 2^31^-1 signal of the Anritsu MP1758A pulse pattern generator (PPG), the Photoline 850 nm modbox 28 Gb/s NRZ transmitter, the λ ~ 1550 nm Oki 43 Gb/s EML module, and Agilent 86100D Digital Communication Analyzer (DCA)-X with a 86118A module were used to measure eye diagrams of the integrated Ge V-PDs at transmission rates of 10 Gb/s to 50 Gb/s. Figure 3(c) shows on-chip measured eye diagrams for 2 μm-*W*_Ge_ Ge V-PD with a 40 μm-ϕ_Ge_, 30 μm-ϕ_Ge_, and 20 μm-ϕ_Ge_ at 25 Gb/s, 35 Gb/s and 50 Gb/s operation, respectively. The measurement of 35 Gb/s and 50 Gb/s eye-diagrams of Ge V-PDs were performed with a Oki λ ~ 1550 nm 43 Gb/s EML module, due to the performance limitation of the λ ~ 850 nm NRZ transmitter used.

**Figure 4:**
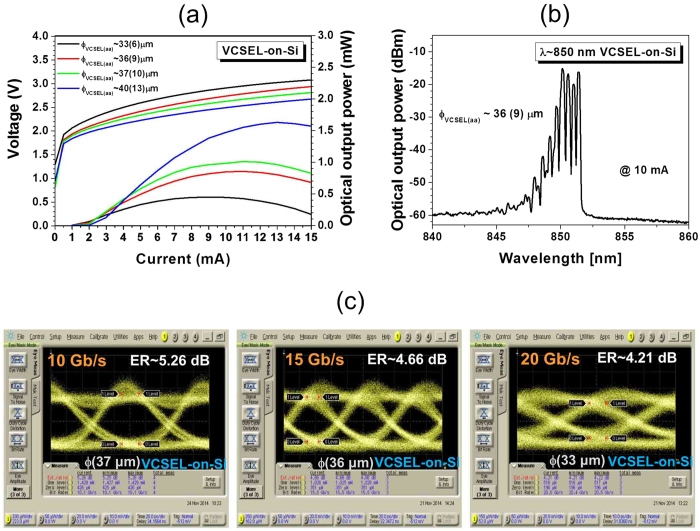
(**a**) Measured L-I and V-I characteristics of monolithic-integrated *VCSELs-on-Si* with 40 μm, 37 μm, 36 μm, and 33 μm mesa-diameters. (**b**) On-chip eye diagram measurements of the monolithic-integrated VCSELs-on-Si with 37 μm, 36 μm, and 33 μm-diameter mesas for 10, 15, and 20 Gb/s operations at 0.6 V_pp_, 0.69 V_pp_, and 0.79 V_pp_ driving and bias currents of 10 mA, 10 mA, and 8 mA, respectively, with NRZ PRBS 2^31^-1 signals.

The monolithic-integrated VCSELs-on-Si were characterized for λ ~ 850 nm with on-chip measurements of the output optical power-current (L-I) and voltage-current (V-I) characteristics at 25 °C. Here, the optical output power was collected on the top of the device by directly coupled multimode fiber. Due to the coupling loss with the fiber, the magnitude of the measured output power was lower than the actual emitted power of the device. Figure 4(a) depicts the measured L-I and V-I characteristics of the VCSELs-on-Si for mesa-diameters of ϕ_VCSEL_ ~ 40 μm, 37 μm, 36 μm, and 33 μm, with active aperture diameter ϕ_aa_ ~ 13 μm, 10 μm, 9 μm, and 6 μm, respectively. The threshold currents are about 1 ~ 2 mA.

Figure 4(b) shows a typical emission spectrum of the VCSEL-on-Si with a ϕ_VCSEL_ ~ 36 μm, at an operating current of ~10 mA, where several transverse modes contribute to the emitted power. The measured capacitance of the fabricated VCSEL-on-Si with a ϕ_VCSEL_ ~ 33 μm and a ϕ_aa_ ~ 6 μm is less than 380 fF near −3 V_DC_. The predicted RC-limit -3dB bandwidth, f_-3dB_ = 1/(2π*RC*), of the fabricated device was ~15.9 GHz. The RF performances of directly-modulated VCSELs-on-Si were characterized by measuring eye-diagrams at high transmission rates. The NRZ PRBS 2^31^-1 signal of the Anritsu MP1800A PPG was combined with a DC bias using a bias-tee, and applied to the VCSELs-on-Si. The modulated output signal from a VCSEL-on-Si was directly coupled to a multimode fiber probe. The light signals were measured with the Agilent DCA with an 86115D 34 GHz optical module.

On-wafer measured eye diagrams at various bit rates from 10 Gb/s to 20 Gb/s for the integrated VCSELs-on-Si, are exhibited in Fig. 4(c). At an operating current of 8 mA and a 0.79 V_pp_ drive, the measured eye-diagram of a VCSEL-on-Si with a 33 μm-ϕ_VCSEL_ exhibits an eye opening up to 20 Gb/s operations. The laser device performance level can be improved with further optimized epi-structures, wafer bonding and device fabrication processes, and we expect that similar device performances of VCSELs-on-Si to those of the VCSELs on original III-V semiconductor substrates can be achieved without much difficulty. For wafer-scale mass production, the integration and fabrication process for VCSEL-on-Si devices can be done more easily with the use of commercial wafer bonder, etc.

### Chip-level optical interconnect experiment

**Figure 5:**
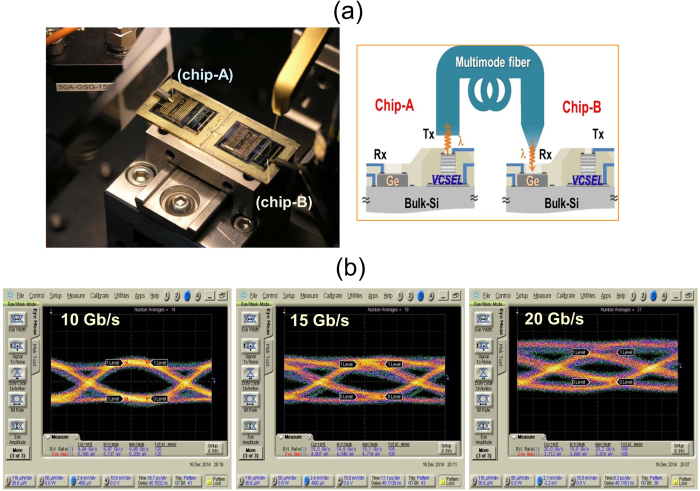
(**a**) The experimental setup and a schematic diagram for chip-level optical interconnect between fabricated TRx chips. The modulated optical signal from a VCSEL-on-Si with 33 μm-ϕ_VCSEL_ on chip-A was collected and transmitted by a multimode fiber directly connected to a Ge-VPD with a 40 μm-ϕ_Ge_ on chip-B. (**b**) The eye diagrams of the transmitted signals for 10 Gb/s to 20 Gb/s data rates detected by the Ge-VPD of chip-B.

An experiment of an inter-chip (chip to chip) λ ~ 850 nm optical interconnection between the fabricated photonic TRx chips was carried out, as shown in Fig. 5. Fig. 5(a) shows the test setup and schematic diagram for a chip-level optical interconnect, where the modulated optical signal of a VCSEL-on-Si of one chip (chip-A) is detected by a Ge V-PD of the other chip (chip-B). Here the NRZ PRBS 2^7^-1 signal of the Anritsu MP1800A PPG was combined with a DC bias using a bias-tee, and applied to a VCSELs-on-Si with 33 μm-ϕ_VCSEL_ on chip-A. The modulated output signals from the VCSEL-on-Si of the chip-A under 8 mA operating current and 0.66 V_pp_ drive were collected and transmitted by a multimode fiber which was directly connected to a Ge-VPD with a 40 μm-ϕ_Ge_ on chip-B. The signals detected by the Ge-VPD, biased at −3 V, were measured by Agilent 86100D DCA-X with an 86118A electrical module. Fig. 5(b) shows the experimental eye diagrams of the transmitted signals at 10 Gb/s to 20 Gb/s data rates from chip-A to chip-B. It shows eye openings up to 20 Gb/s where the power consumption for the inter-chip interconnect was about 1.2 pJ/bit.

This single-chip photonic TRx scheme can simultaneously increase the level of photonic integration and reliability, as well as performance levels in the applied systems, and has many advantages for realistic, implementable, cost-effective applications. With properly designed VCSEL epi-structures and optimizations of VCSEL-on-Si device fabrication processes, the single-chip transceivers are capable of high performance up to ~50 Gb/s^47^,^48^,^49^,^50^,^51^,^52^,^53^. Moreover, since the effective absorption wavelength range of germanium is wide, from λ ~650 nm to λ ~ 1600 nm, any type of vertical emitting lasers within this wavelength range, for example, 980 nm, 1060 nm, 1310 nm, or 1550 nm and so on, can be used as the counterpart chip-level light source in the proposed single-chip TRx scheme for various types of applications.

**Figure 6:**
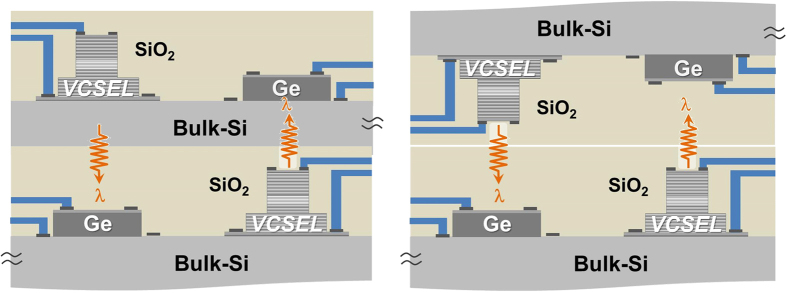
Schematic examples of 3D stacking of silicon multichips with vertical optical I/Os. The dielectric waveguide structures with Mux/DeMux devices also can be included on the top dielectric of vertical optical I/Os for WDM functionality of the silicon chips.

In particular, this complete chip-level optical I/Os on a bulk-silicon chip approach is suitable for 3D interconnect applications in stacked multichip systems by configuring the TRx devices accordingly. Figure 6 illustrates examples of 3D optical interconnects based on the proposed vertical photonic I/O scheme. By using monolithic-integrated surface- or bottom-emitting lasers with the appropriate emitted laser wavelength λ > ~ 1 μm required for transparency through the bulk-silicon substrate, and proper arrangement (configuration) of vertical photonic TRx devices on silicon chips, 3D optical interconnects are possible, which can enhance the performance and effectively reduce the large numbers of normal through-silicon-vias (TSVs) required for 3D interconnects in stacked dies for 3D-ICs^58^.

The monolithic integration of multiple functional optical components on the same wafer can cost-effectively increase the performance of PICs. WDM functionality can be also included in the proposed scheme by integrating multi wavelength VCSELs-on-Si^59^ and passive devices defined with dielectric waveguides, such as SiN_x_^60^, on the top (or bottom) passivation layer of a vertical photonic TRx chip, and this can effectively reduce the I/O count via multiplexing.

## Discussion

In this article, we have presented and demonstrated a new photonic integration scheme. The single-chip photonic transceiver is based on monolithic-integrated vertical photonic TRx devices on the same bulk silicon substrate. The demonstrated approach solves the substrate compatibility issue with bulk Si CMOS microelectronics and the chip-level low-power consumption compact light source issue, simultaneously. A prototype of the single-chip photonic transceiver was capable of a chip-level λ ~ 850 nm optical interconnect. The monolithic-integrated vertical-illumination type Ge-on-Si photodetectors exhibited device performance up to 50 Gb/s, and the monolithic-integrated light source, directly-modulated VCSELs-on-Si on the same bulk silicon substrate exhibited device performance up to 20 Gb/s. This study experimentally demonstrated a viable chip-level photonic I/O platform operating at high data rates. The fabricated prototype confirmed that the proposed single-chip photonic transceiver scheme can be used as a compact, reliable, low-energy, chip-level platform, to realize monolithic photonic integrated circuits on the same bulk Si substrate with CMOS electronics. The proposed I/O scheme holds great potential for large-scale cost-effective mass production with minimal extra cost and reliable operation, in future silicon-based optical communications.

## Methods

### Device fabrication

To fabricate the single-chip transceiver, Ge V-PDs, the photonic Rx side on a silicon chip were processed first. Phosphorous implantation was performed with a doping level of 5 × 10^19^/cm^3^ in predefined regions for V-PDs on a 6” Si (100) wafer in the I-line lithography node. After SiO_2_ layer deposition on the wafer, the lithographed patterns for PD mesa windows with various diameters from 20 μm to 90 μm were defined by dry etching process. Ge epitaxial layers were selectively grown in the opened mesa window patterns by RPCVD. A 0.1 μm-thick Ge seed layer was grown at 380 °C, followed by a Ge absorption layer grown at 650 °C without additional thermal annealing process on the patterned windows. Germanium up to ~4 μm thickness can be grown with good quality. A cap layer of 0.1 μm-thick in-situ boron-doped poly-silicon with a doping level of 5 ×1 0^20^/cm^3^ was deposited on top of the Ge absorption layer at 750 °C, providing a heavy and uniformly doped layer without ion implantation for the p-type contact. The devices were fabricated with CMOS compatible processes of photolithography, etching, passivation, metallization and alloying with Ti/TiN/Al_1%Si/TiN. After integration of the Ge V-PDs, a SiO_2_ layer was deposited on the fabricated chip for the protection of the integrated V-PDs. For the monolithic integration process of laser light source on the silicon chip, the fabrication of the optical transmit side (Tx) was started by wafer bonding with a substrate lift-off VCSEL epitaxial film [65,66] on the dedicated region, which was formed by opening a window for the VCSEL epi-chip bonding area, by dry etching process. It is possible to use any type of designed VCSEL epi-structures with emitting wavelengths, which are within the effective absorption wavelength range of germanium between λ ~ 650 nm and λ ~ 1600 nm. Here, we used a shorter wavelength VCSEL epi-structure, designed for λ ~ 850 nm, containing 3 GaAs quantum wells with an etch-stop layer grown on a GaAs substrate. The spin-on glass material (SOG) was coated on a silicon chip for bonding of a small piece of the VCSEL epi-wafer in the opened window area of the silicon chip. The bonding process was performed with heat and pressure in a homemade chamber. The separation of the epitaxial film from the GaAs substrate on which it was grown was accomplished by etching process with NH_4_OH solution. After removal of the GaAs substrate, the device fabrication process was carried out for the VCSEL epitaxial film on silicon, including photolithography with a contact-aligner, top metallization with Au/Ge/Ni, RIE and wet etching processes to define various diameter (ϕ_VCSEL_) mesas, and an oxidation process to form the device active aperture with ϕ_aa_ diameter. Planarization with SU-8 3000 for ~10 μm tall device mesas, and bottom metallization with Ti/Pt/Au for VCSELs-on-Si were performed. Here, it is possible to replace the SU-8 planarization process with an SiO_2_ passivation process or polyimide process. The window opening process for the protected Ge V-PDs during the VCSELs-on-Si fabrication process was performed for the device characterization. A SiO_2_ passivation layer can also be used as anti-reflection coating, which was optimized for λ ~ 850 nm in this case. As mentioned before, extension to longer wavelength VCSELs-on-Si, for example, λ ~ 1310 nm or λ ~ 1550 nm range, can be straight forward by using properly-designed VCSEL epi-wafers based on InP substrates and low-temperature plasma wafer bonding process, etc. For wafer-scale mass production, the integration and fabrication process for VCSEL-on-Si devices can be done more easily with a commercial wafer bonder.

## DC measurements

### I-V measurements

I-V characteristics of the Ge-on-Si photodetectors and VCSELs-on-Si were measured with an HP 4156A Parameter analyzer. Photocurrent under illumination with a ~850 nm light from a Thorlab multichannel fiber coupled laser source was measured with a lensed multi-mode fiber directly-coupled to the top of the device, and a HP Parameter analyzer.

### L-I & emitted spectra measurements

For the optical output power (L)-current (I) measurements of VCSELs-on-Si, a VCSEL-on-Si was biased with a Keithley Source Measurement Unit 2440, and the emitting light was collected by the multimode fiber directly-coupled to the top of the device, and measured with an Agilent A8163A Lightwave multimeter with A81635 power sensor module. The emitted spectra of a VCSEL-on-Si biased with a Keithley Source Measurement Unit 2440 were measured with HP 86143B optical spectrum analyzer.

### Capacitance measurements

The capacitance of the fabricated device was measured with an Agilent E4980A Precision LCR meter.

## RF measurements

### Impulse response measurements

Pulsed light signals from a Menlo systems TC-1550 femtosecond pulse laser were fed to a Ge-on-Si PD biased with a bias-T and a Keithley Source Measurement Unit 236, by a single-mode lensed fiber directly coupled on top of the device. The impulse response signals of a device in time domain were measured with an Eigenlight power monitor 420 WDM optical attenuator and an Agilent DCA-X 86100D digital communication analyzer with 86118A 70 GHz electrical head module. The measured impulse response data in time-domain was Fourier-transformed to the data in frequency domain, and was de-embedded with contributions from the connecting RF cables and probe. From the frequency response curve, -3 dB bandwidth was determined.

### Eye diagram measurements

The eye diagrams of the integrated Ge V-PDs at a wavelength of 850 nm up to 30 Gb/s, were measured with the non-return-to-zero (NRZ) pseudo-random bit sequence (PRBS) 2^31^-1 signal of the 4 channel Anritsu MP1800A with MU181020A 12.5 Gb/s pulse pattern generator (PPG), MP1821A 50 Gb/s Mux, Agilent E8257D RF signal generator, the Photoline 850 nm modbox 28 Gb/s NRZ transmitter, a Keithley Source Measurement Unit 236 and bias-T by Agilent DCA-X 86100D digital communication analyzer with 86118A 70 GHz electrical head module. Because the λ ~ 850 nm Photoline transmitter signal quality was limited beyond 28 Gb/s, the measurement of eye-diagrams from 30 Gb/s to 50 Gb/s were performed with an Oki λ ~ 1550 nm 43 Gb/s EML module. To measure eye diagrams of the integrated VCSELs-on-Si biased with a bias T and a Keithley Source Measurement Unit 236, the NRZ PRBS 2^31^-1 signal of the 4 channel Anritsu MP1800A with MU181020A 12.5 Gb/s PPG with MP1821A 50 Gb/s Mux and Agilent E8257D RF signal generator was applied to the VCSEL-on-Si device. The modulated light was collected by a multimode fiber directly-coupled to the top of the device, and was measured by an Agilent DCA-X 86100D with an 86115D 34 GHz optical module.

## Additional Information

**How to cite this article**: Kim, G. *et al.* Single-chip photonic transceiver based on bulk-silicon, as a chip-level photonic I/O platform for optical interconnects. *Sci. Rep.* **5**, 11329; doi: 10.1038/srep11329 (2015).
